# Seasonal Efficiency of Supplemental LED Lighting on Growth and Photomorphogenesis of Sweet Basil

**DOI:** 10.3389/fpls.2021.609975

**Published:** 2021-04-06

**Authors:** Jan Andreas Solbach, Andreas Fricke, Hartmut Stützel

**Affiliations:** Vegetable Systems Modelling Section, Institute of Horticultural Production Systems, University of Hannover, Hannover, Germany

**Keywords:** Basil (*Ocimum basilicum* L.), light dose-response curves, light interception, light use efficiency, photomorphogenesis, supplemental lighting, light emitting diode (LED), far-red light

## Abstract

For decisions on supplemental lighting a quantitative knowledge of the plants' responses to light under varying conditions is fundamental. In this study, we developed light dose-response curves of growth and morphological traits for *Ocimum basilicum* L. and examined the effects of light color (blue, red, and white plus far-red) and natural environment (season) on these curves. Four greenhouse experiments were conducted throughout the year to determine the efficiencies of the light regimes on growth and their effects on plant morphology. A special aspect was the photosynthetic efficiency of far-red light. Linear and monomolecular relationships were found for the relationships between plant traits and supplemental light dose. Traits related to biomass productivity increased linearly with light dose whereas some morphological characters showed a saturation behavior. Red light and white plus far-red light were more efficient in plant dry weight production than blue light, and the plants adapted differently to the light qualities: higher biomass under red light was related to a plant architecture more favorable for light capture, i.e., taller plants and bigger leaves. White plus far-red light, on the other hand, increased leaf mass per area (LMA) and light use efficiency (LUE). Blue light resulted in lowest plant light interception and LUE. Considering photosynthetic effects of near-infrared light (PPFD_800_, 400–800 nm) instead of photosynthetic photon flux density (PPFD_700_, 400–700 nm) led to strongly reduced efficiencies. Traits related to photosynthesis such as dry weight, LMA and LUE were particularly affected by PPFD_800_. There were no interactions between the efficiencies of the different light colors and the seasons. Efficiencies of all light regimes were significantly lower during summer compared to spring and winter. Higher dry weight production during summer compared to winter and spring were a consequence of increased light interception rather than changes in LUE. The observed differences in seasonal efficiencies were directly linked to the amount of natural light present as indicated by changes in the ratio of supplemental to natural light.

## Introduction

Growth and morphology of plants are strongly influenced by the light environment under which they are grown. Light spectrum, light dose, photoperiod, and the growing period are major determinants of the plant's adaptation to the environment (Goto, 2003). In general, plants grow under natural daylight that strongly varies in light spectrum and intensity depending on the weather, time of day, season and atmospheric conditions. For example, an overcast sky leads to increased proportions of blue light, whereas the ratio of red to far-red (R/FR) has been shown to vary little with weather conditions and season and is roughly 1.2 in natural daylight. Changes in R/FR occur during sunrise and sunset as well as when the light penetrates the plant canopy (Smith, 1982). A reduction in R/FR induces photomorphogenetic effects, known as shade-avoidance responses, that are usually characterized by a rapid elongation of stems and leaves (for comprehensive reviews on shade-avoidance see: Franklin and Whitelam, 2005; Vandenbussche et al., 2005; Franklin, 2008).

Red light (600–700 nm) is considered the most efficient in driving plant photosynthesis (e.g., McCree, 1972; Hogewoning et al., 2012) although the “red light syndrome” is commonly observed in studies under controlled environments. The syndrome is characterized by reduced plant growth and development (e.g., Goins et al., 1997; Yorio et al., 2001) due to decreased photosynthetic capacity, leaf thickness and nitrogen. The addition of blue light (400–500 nm) may alleviate the symptoms (Hogewoning et al., 2010; Ouzounis et al., 2016; Trouwborst et al., 2016). Blue light is involved in a number of physiological processes including the development of sun-type chloroplasts (Lichtenthaler et al., 1980), chloroplast movement (Banaś et al., 2012) and stomatal movement (Shimazaki et al., 2007). Blue light mediates stomatal opening, but this may be reversed by adequate amounts of green light (Frechilla et al., 2000; Talbott et al., 2002, 2006). Furthermore, it has been shown that green light (500–600 nm) may drive photosynthesis more efficiently than red light in white background light (Terashima et al., 2009). Moreover, recent studies suggest that far-red photons (701–750 nm) may drive photosynthesis equally efficiently as photons in the PAR (400–700 nm) region (Zhen and Bugbee, 2020a,b).

As previously pointed out (Hemming, 2011), most LED lighting studies investigating plant responses to light quality were conducted in controlled environments under completely artificial light, rather than under greenhouse conditions with natural background radiation. Sunlight is composed of different wavelengths ranging from ultraviolet to infrared and approximately equal proportions (~20–25%) of blue, green, and red photons (Smith et al., 2017). Results from artificial environments may therefore not always be directly transferable to greenhouse production conditions although they certainly help to broaden our understanding of the principles of plant responses to light. On the other hand, since light intensity and spectra continuously fluctuate in natural sunlight, light quality, and dose effects of supplemental LED lighting may vastly vary in size which makes the interpretation of results and the comparison of studies difficult. Hence, a clear distinction between light intensity or spectral effects is often not possible. Several studies reported no quality effects of supplemental LED lighting during greenhouse production of ornamentals and vegetables (Hernández and Kubota, 2012, 2014; Bergstrand and Schüssler, 2013). Bergstrand and Schüssler (2013) supposed that during greenhouse production the effect of supplemental light quality depends on the amount of natural radiation present, and Hernández and Kubota (2012, 2014) concluded that the natural light environment already meets quantitative blue light requirements. Recent findings showed that the lack of blue light in the supplementing light source did not induce symptoms of the “red light syndrome” indicating that there might be such a blue light threshold as mentioned by Hernández and Kubota (2012, 2014) although adding increased proportions of blue light increased biomass and yield of tomato to an optimum under greenhouse conditions (Kaiser et al., 2019).

These inconsistent and partially contradictory results of the effects of supplemental LED lighting under solar background make it difficult to predict plant responses to the applied supplemental light spectrum. In particular, little is known about the influence of seasonally varying natural light environments on the effects of supplemental LED lighting in greenhouse production.

Thus, the present study aims to investigate the following questions:

1. How does light dose affect the efficiency of supplemental radiation for plant growth and its effects on plant morphology?
2. Do trait value dose-responses follow the same pattern among light colors?
3. How does the background radiation (season) affect the efficiency of supplemental lighting?
4. How do efficiencies change when near-infrared is considered photosynthetically active, i.e., when PPFD_800_ (400–800 nm) is used instead of PPFD_700_ (400–700 nm)?

To answer these questions, we analyzed the efficiency of supplemental LED lighting under greenhouse conditions on growth and morphological traits of sweet basil under blue, red and white plus far-red LEDs as dependent on the level of supplemental and natural light.

## Materials and Methods

### Plant Material and Growing Conditions

Four greenhouse experiments were conducted at the Institute of Horticultural Production Systems, Leibniz University Hannover, Germany (lat. 52°23′N, long. 9°39′E) in 2018. The first trial was carried out from February 8 to March 19 (Late Winter), the second from March 13 to April 16 (Mid Spring), the third from May 22 to June 27 (Early Summer) and the fourth from August 2 to September 11 (Late Summer). Growing parameters and environmental conditions that prevailed during the experiments are shown in Table 1. In each trial, *Ocimum basilicum* L. cv. “Edwina” seeds (Enza Zaden Beheer B.V., Enkhuizen, Netherlands) were sown in 10-cell trays (5 cm H × 4.5 cm W × 4.5 cm L per hole) in a fertilized peat substrate (Potgrond H, Klasmann-Deilmann GmbH, Geeste, Germany) and germinated in a greenhouse without supplemental lighting. Each tray included one plant per cell. When the seedlings emerged at the substrate surface, they were subjected to the supplemental lighting treatments (see next section). Seeds that did not germinate were replaced by transplants to restock the canopy. Plants were irrigated and fertigated when necessary with a 2 g L^−1^ concentrated nutrient solution (Ferty®Mega 2, Planta Düngemittel GmbH, Regenstauf, Germany). Greenhouse day/night temperatures were set to 20/16°C and ventilation opened at 24°C.

**Table 1:** Overview of the growing conditions in the greenhouse.

	**Experiment (Season)**							
	**Late Winter**		**Mid Spring**		**Early Summer**		**Late Summer**	
**Harvest point**	**Int^**u**^**	**Final**	**Int**	**Final**	**Int**	**Final**	**Int**	**Final**
Growing period^v^ [d]	18	32	20	28	15	29	14	33
Mean air temperature [°C]	19.3		20.6		24.4		24.1	
Temperature sum^w^ [°Cd]	159.4	290.2	191.4	277.5	228.6	401.4	202.3	446.3
Mean relative humidity [%]	34.8		43.8		53.9		49.4	
Mean natural DLI^x^ [mol PAR m^−2^ d^−1^]	7.1		9.8		15.7		13.8	
Cumulated light sum^y^ [mol PAR m^−2^]	127.7	226.8	186.1	274.5	254.0	454.9	217.8	456.6
SL/NL^z^	1.72		1.24		0.78		0.88	

^u^*Int, Intermediate; ^v^Counted from the start of the supplemental lighting treatment; ^w^Calculated following McMaster and Wilhelm (1997). T_base_ for basil was taken as 11°C (Walters and Currey, 2019); ^x^DLI, Daily light integral; ^y^Calculated by cumulating DLI's each day from the start of the supplemental lighting treatment; ^z^SL/NL, Ratio of supplemental light to natural light directly under the LEDs calculated over the growing period*.

### Supplemental Lighting Setup and Treatments

**Figure 1:**
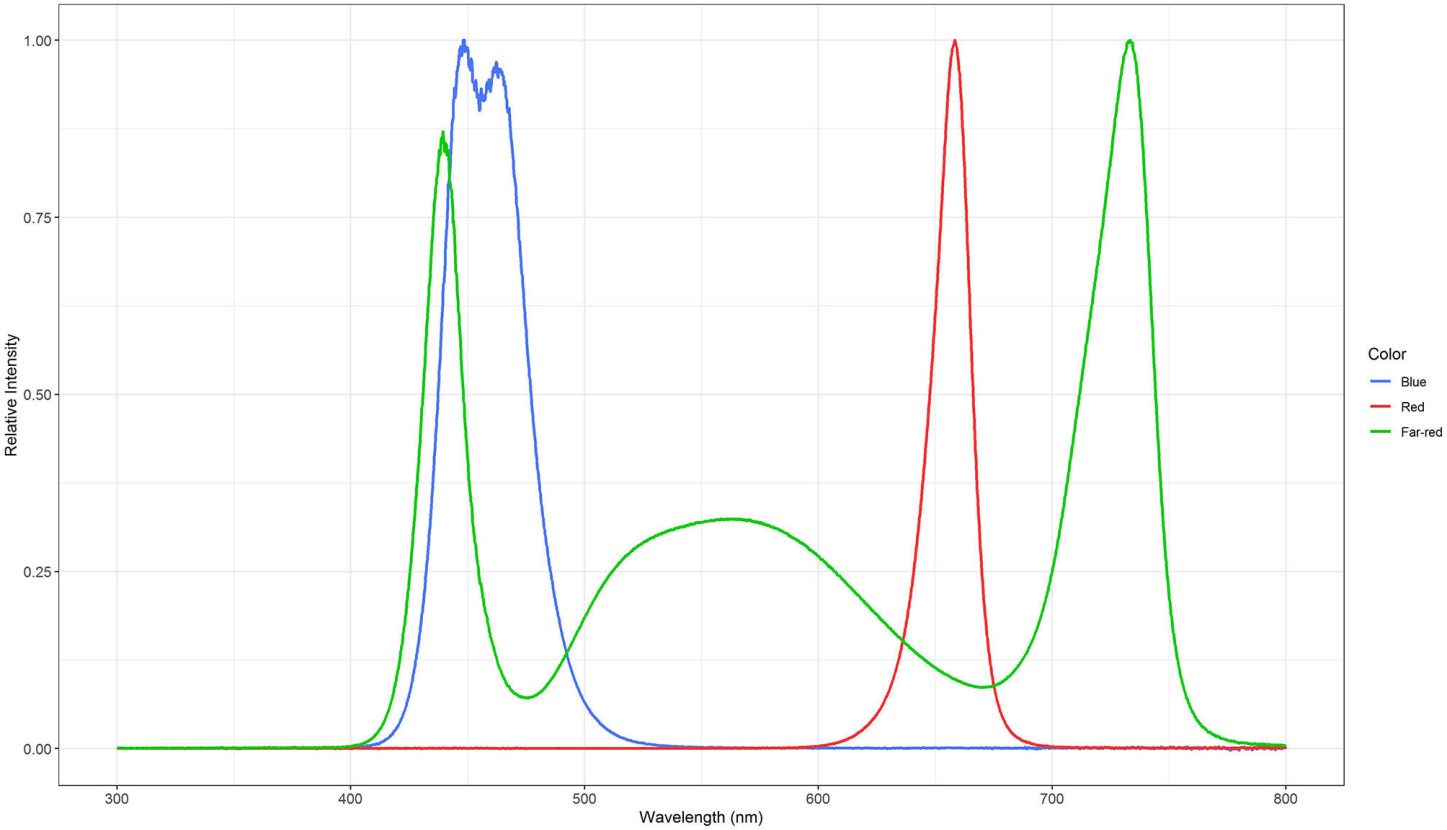
Normalized spectral distributions of the blue (440 + 470 nm), red (660 nm), and far-red (730 nm) light treatments. The far-red light treatment included white light (400–700 nm) to ensure the same PPFD_700_ in all treatments.

**Figure 2:**
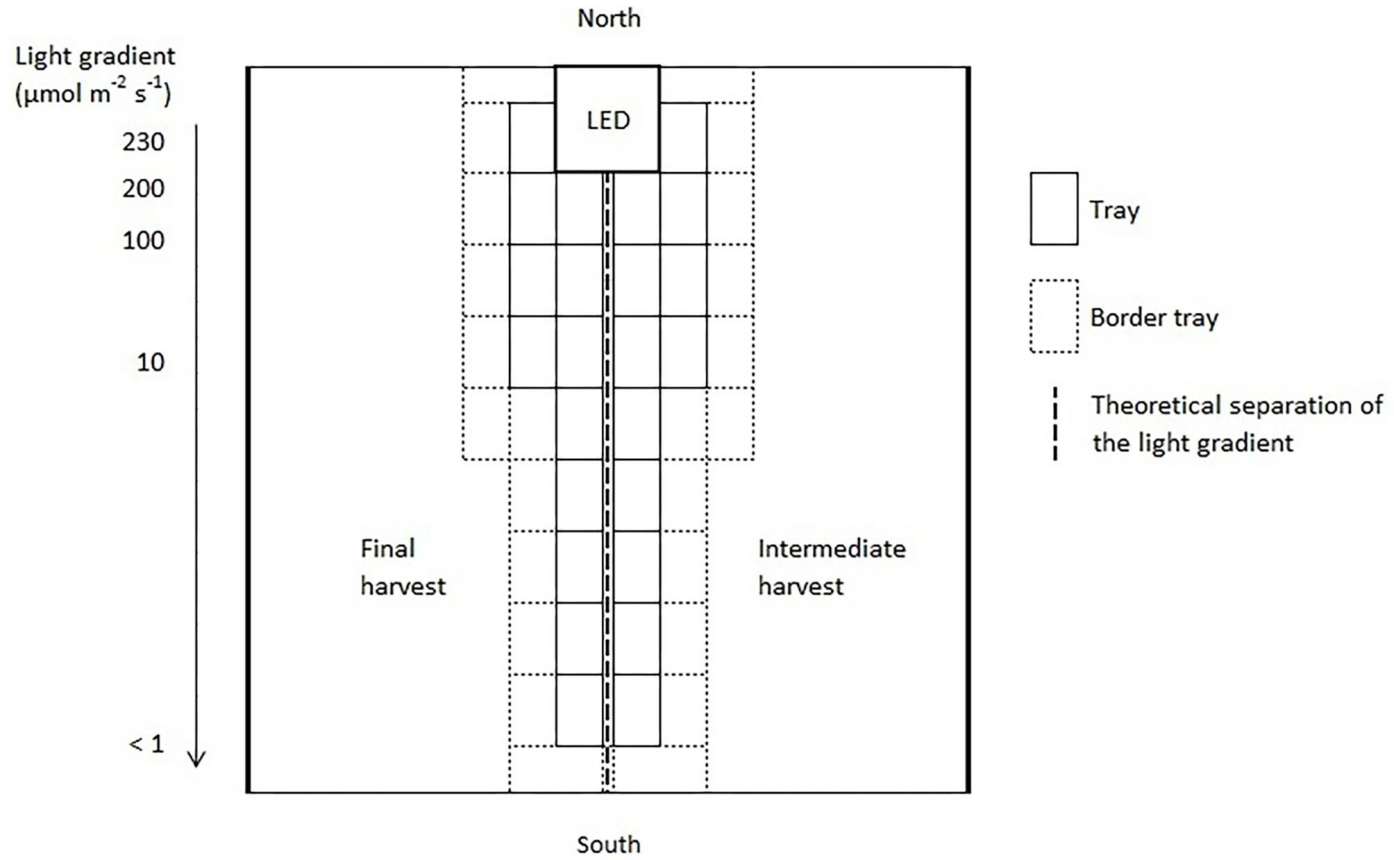
Schematic representation of one light quality treatment of the supplemental lighting setup. The positions of the light quality treatments and harvests were randomized in each replication and season.

Three light quality treatments were arranged in a randomized complete block design on two benches (4.8 m × 2 m), each of them was evenly divided into three compartments (1.6 m × 2 m). On each bench, a full repetition of the experiment was carried out. The distance between the two benches was 2.7 m and the compartments were separated at the eastern and western edges through 2 m × 0.40 m double layered 0.08 mm black plastic films (Lux Baufolie, Emil Lux GmbH & Co. KG, Wermelskirchen, Germany) to eliminate light pollution among treatments and to interfere as little as possible with the natural radiation. Each compartment included one LED lamp (LED-KE 300, DH Licht GmbH, Wülfrath, Germany) that could be adjusted in light color and intensity via a software (VisuSpectrum v2.0, RAM GmbH Mess- und Regeltechnik, Herrsching am Ammersee, Germany). Light quality treatments were: blue (440 + 470 nm), red (660 nm), and far-red (730 nm, Figure 1). The far-red treatment additionally included white light (6,500 K, 400–700 nm) to ensure the same PPFD (PPFD_700_, 400–700 nm) in all treatments and resulted in a red to far-red ratio (calculated according to Franklin, 2008) of 0.1. We additionally defined photon flux density (PPFD_800_, 400–800 nm) of the far-red light treatment. LEDs were turned on at 4 a.m. and turned off at 10 p.m. (18 h day/6 h night). The LEDs were mounted centrally in 0.60 m distance from the bench's surface at the northern edges of each compartment to create a continuous supplemental light gradient ranging from ~230 μmol PPFD_700_ m^−2^ s^−1^ directly below the LED lamp to <1 μmol PPFD_700_ m^−2^ s^−1^ at the edge of the compartment. In each light color treatment, trays with a total of 240 basil plants were arranged in a straight line from beneath the LED lamp to the end of the treatment over the whole compartment to cover the entire length of the light gradient. A distinct specific supplemental light dose was assigned to each plant within the gradient. Furthermore, two harvest dates, namely intermediate and final harvest, were arranged within the gradient by splitting it in the middle (120 plants in both harvests), and thereby mirroring the gradient on two sides (Figure 2). The positions of the light quality treatments and harvests were randomized in each season. In addition, border rows were arranged to eliminate edge effects. Border rows were moved toward the center to close the canopy after the intermediate harvest.

### Light Measurements and Unit Conversion

Greenhouse light transmission was determined to be 0.55 by relating PPFD_700_ inside the greenhouse to outside PPFD_700_. PAR quantum sensors connected to a data logger (LI-1100 DataLogger, LI-COR Inc., USA) were used to collect the inside light data at plant height and natural radiation was recorded by a weather station next to the greenhouse throughout the experiments. An average of five days was taken to calculate the transmission factor. PPFD_700_, PPFD_800_ and light spectra of the LED treatments were measured under exclusion of natural radiation with a spectroradiometer (USB4000, OceanInsight, formerly OceanOptics, USA) equipped with a 3,900 μm optical fiber and a cosine corrector (CC-3-UV-S, OceanInsight, formerly OceanOptics, USA). Quantum flux (μmol PAR m^−2^ s^−1^) was converted into daily light energy integral (MJ PAR m^−2^ d^−1^) integrating over the light period and using a conversion factor of 0.219 (Thimijan and Heins, 1983).

### Growth and Morphological Measurements

Data were collected at two harvest points (Table 1) from individual plants. Shoots were cut at the soil surface and partitioned into stems and leaves. Fresh weights of the two organs were taken and subsequently leaf and stem samples were dried in an oven (TU-2, Heraeus Holding GmbH, Hanau, Germany) at 70°C for at least 72 h to determine dry weights. Leaf area was determined with a leaf area meter (LI-3100C, LI-COR Inc., USA) prior to leaf drying. Leaf mass per area (LMA, leaf dry weight divided by leaf area) and stem to leaf ratio (stem-leaf ratio, stem dry weight divided by leaf dry) were calculated. Furthermore, hypocotyl, epicotyl, and internode lengths were measured with a ruler. Plant height was defined as the sum of these lengths.

### Estimation of Light and Energy Use Efficiency

The amount of daily absorbed PPFD_700_ and PPFD_800_ (*Q*_*daily*_, MJ m^−2^ d^−1^) of a plant was calculated as described by Monsi and Saeki (2005) following Beer–Lambert's law, respectively:

(1) $$\begin{array}{r} Q_{daily}=I\times \left(1-e^{-k\times LAI}\right) \end{array}$$

where *I* is the daily recorded PPFD_700_ and PPFD_800_ (MJ m^−2^ d^−1^) above the plant, respectively, *LAI* is the leaf area index (m^2^ leaf area per m^2^ ground area, the ground area is defined here as 0.002025 m^2^ per plant^−1^) and *k* is the light extinction coefficient, assumed as 0.8 for basil. *I* is the sum of supplemental and natural PPFD_700_ or PPFD_800_ inside the greenhouse. As leaf areas could only be measured at the two harvests, leaf areas between the start of the supplemental lighting treatment and harvest 1, and between the two harvests were interpolated on the basis of temperature sum [°Cd, calculated following McMaster and Wilhelm (1997); *T*_base_ for basil was taken as 11°C (Walters and Currey, 2019)] based on three data points: the measured leaf area at intermediate and final harvest, and the zero point at the start of the supplemental light treatment (day 0). The three data points were log-transformed and a linear regression was fitted to the data to estimate leaf areas for each day starting from the beginning of the supplemental lighting treatment. Subsequently, logarithmic estimated leaf areas were back transformed to follow an exponential function. In experiment 3 and 4, two linear regressions (the first based on zero point and intermediate harvest, and the second based on intermediate and final harvest) were fitted to estimate daily leaf areas instead because estimation on three data points deviated >25% from the measured data points.

The total amount of absorbed PPFD_700_ and PPFD_800_ (*Q*_*total*_, MJ m^−2^) of a plant was then calculated by accumulating *Q*_*daily*_ from the beginning of the supplemental lighting treatment until the intermediate and final harvest, respectively.

The light use efficiency (*LUE*, g MJ^−1^) of a canopy was expressed as the quotient of the dry weight (*DW*, g) of the plant to *Q*_*total*_ at intermediate and final harvests:

(2) $$\begin{array}{r} LUE=\frac{DW}{Q_{total}} \end{array}$$

Potential energy use efficiency (*EUE*, g MJ^−1^) was calculated by dividing the dry weight of a plant produced by the supplemental light only (*DW*_*SL*_, g m^−2^) by the supplemental light received by it (*SL*, MJ m^−2^) and multiplying it by the electrical conversion efficacy (μ):

(3) $$\begin{array}{r} EUE=\frac{DW_{SL}}{SL}\times \mu \end{array}$$

where conversion efficacies of blue, red, and white plus far-red were 0.37, 0.63, and 0.37 MJ MJ^−1^, respectively. Conversion efficacies were calculated by relating the total light output of the LED lamp to its power consumption for each light color.

### Statistical Analysis

Linear regressions of growth and photomorphological responses on light intensity were calculated to derive regression coefficients (i.e., slopes and intercepts) based on 120 biologically distinct plants, each with a specific light dose, for each replication of each treatment separately. Response and explanatory variables were log and square root transformed, respectively, prior to calculation of regression lines to approximate normal distribution and homogeneity of data.

Slopes and intercepts were then evaluated with a linear mixed-effects model

(4) $$\begin{array}{r} y={\left(c+s+h\right)}^{2}+RS+RSC+RSCH+e \end{array}$$

where *y* is the response (i.e., slopes and intercepts), *c* is the supplement light color (blue, red, and white plus far-red), *s* is the season (Late Winter, Mid Spring, Early Summer, and Late Summer), *h* is the harvest point (intermediate and final), and *RS, RSC*, and *RSCH* describe the randomization units which include the replication per season, replication per season per color and replication per season per color per harvest, respectively, and *e* is the residual error. Fixed effects are presented in lower case letters and random effects in capital letters. Analyses were conducted in RStudio (RStudio Team., 2016), an integrated development for R (R Core Team., 2020), using *lmer* function of the lme4 package (Bates et al., 2015) to fit the linear mixed-effects model to the data. The *anova* function was used to examine differences between the fixed effects as well as their two-way interactions (*p* < 0.05). Estimated marginal means were computed based on the fitted model using the *emmeans* function of the emmeans package (Lenth, 2020). When the data showed a saturating trend, monomolecular functions were fitted and compared to the linear model using a *F*-test.

## Results

### Effects of PPFD_700_ and Light Color on Plant Growth and Morphology

**Figure 3:**
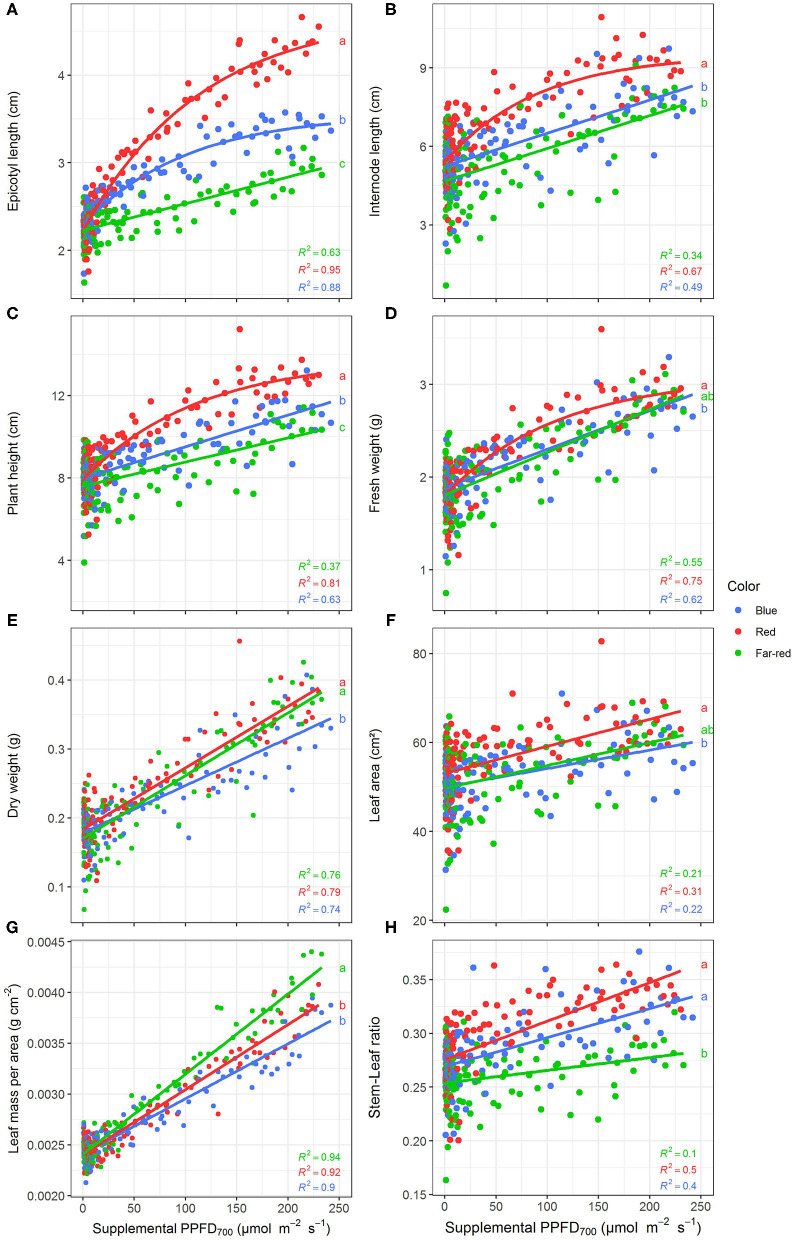
Effect of supplemental PPFD_700_ (400–700 nm) with different spectra on epicotyl length **(A)**, total internode length **(B)**, height **(C)**, fresh weight **(D)**, dry weight **(E)**, leaf area **(F)**, leaf mass per area **(G)**, and stem-leaf ratio **(H)** of *Ocimum basilicum* L. cv. “Edwina” cultivated under greenhouse conditions. Different lower-case letters indicate significant differences in slopes between colors (*p* < 0.05). Linear or monomolecular regression lines of each light color are indicated. Regressions are based on 120 plants per treatment, each with a specific light dose. Each data point shows an average of the four seasons, two harvest points and two experimental repetitions.

**Figure 4:**
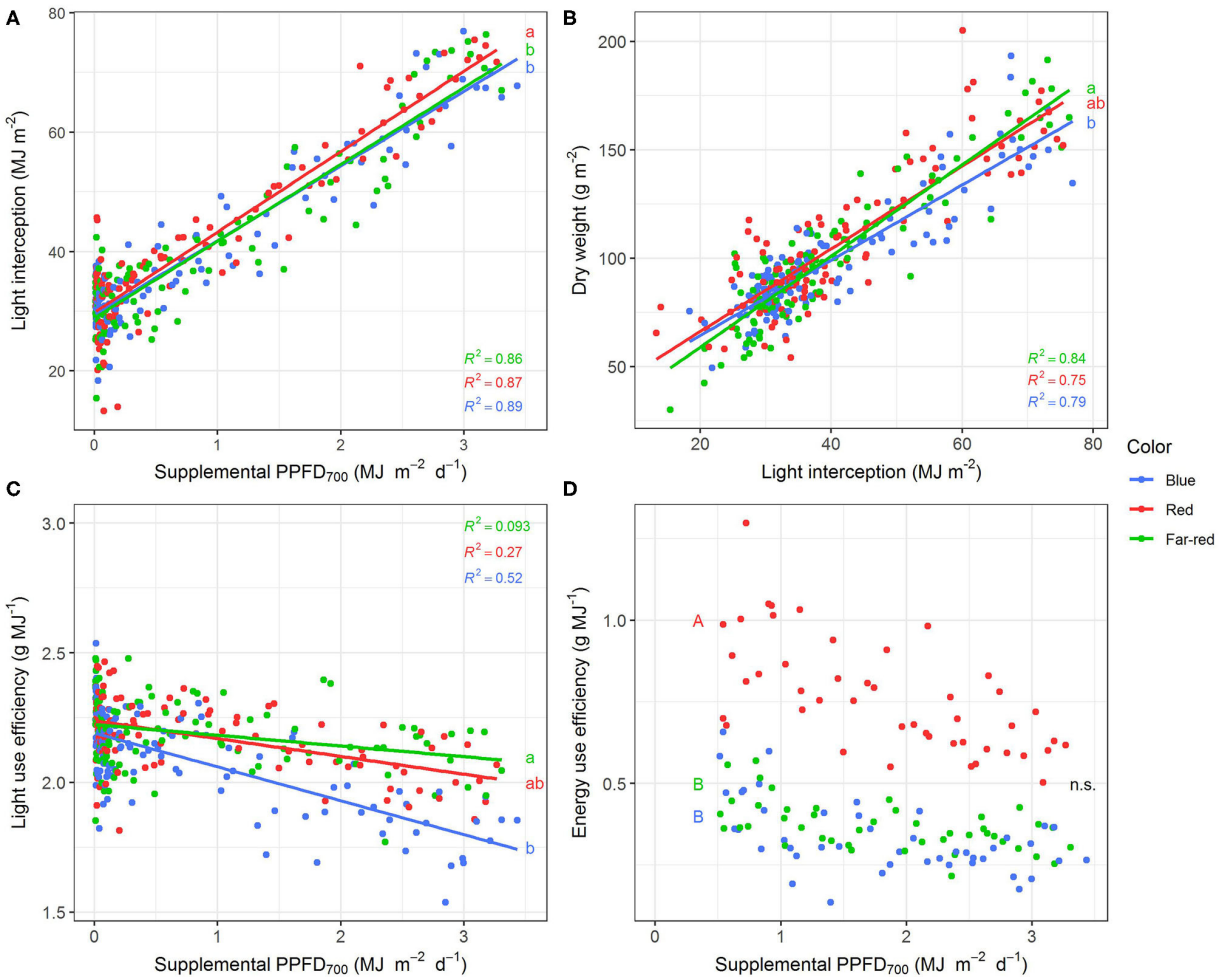
Effect of supplemental PPFD_700_ (400–700 nm) with different spectra on light interception **(A)**, dry weight **(B)**, light use efficiency **(C)**, and energy use efficiency **(D)** of *Ocimum basilicum* L. cv. “Edwina” cultivated under greenhouse conditions. Different lower-case and upper-case letters indicate significant differences in slopes and intercepts between colors (*p* < 0.05; n.s., non-significant), respectively. Intercepts of **(A–C)** were not significant. Linear regression lines of each light color are indicated. Regressions are based on 120 plants per treatment, each with a specific light dose. Each data point shows an average of the four seasons, two harvest points and two experimental repetitions.

All investigated efficiencies (slopes) of growth and morphological traits were significantly (*p* < 0.05) affected by light quality but there were no interactions between light color and season as well as light color and harvest point (Supplementary Table 1). Positive linear or monomolecular relationships were found between all plant parameters and supplemental light intensity, except for LUE and EUE which were negatively correlated with supplemental PPFD_700_ dose (Figures 3, 4). Monomolecular relationships indicating a saturating light response were mainly observed under red light and on plant traits resulting from expansive processes such as epicotyl and internode elongation (Figures 3A,B), rather than traits related to plant productivity which showed linear responses to supplemental light throughout (Figure 3E).

Red light stimulated the elongation of the plant axes (i.e., epicotyl, internode length, and plant height, Figures 3A–C) more than far-red and blue light. For shoot dry weight production, red and white plus far-red light had steeper slopes, i.e., were more efficient (Figure 3E) than blue light. The high dry weight production under red light was associated with the highest leaf area (Figure 3F) and high stem-leaf ratio (Figure 3H) which were more favorable for light interception (Figure 4A). On the other hand, plants grown under white plus far-red light invested a higher proportion of dry weight into leaves (Figure 3H). Increasing LMA (Figure 3G) resulted in a better photosynthetic utilization of the incident light, i.e., higher LUE (Figures 4B,C) indicating a higher photosynthetic capacity of the leaves. Blue light was least efficient for plant dry weight production (Figure 3E) which was due to a low light interception (Figure 4A) accompanied by a poor LUE (Figures 4B,C). Electrical energy use efficiency (EUE) was about two-fold higher for the plants grown under red light compared to the other two treatments (Figure 4D) and mainly related to differences in electrical energy conversion efficacy among light colors.

### Seasonal Influences on the Efficiencies of Supplemental LED Lighting

**Figure 5:**
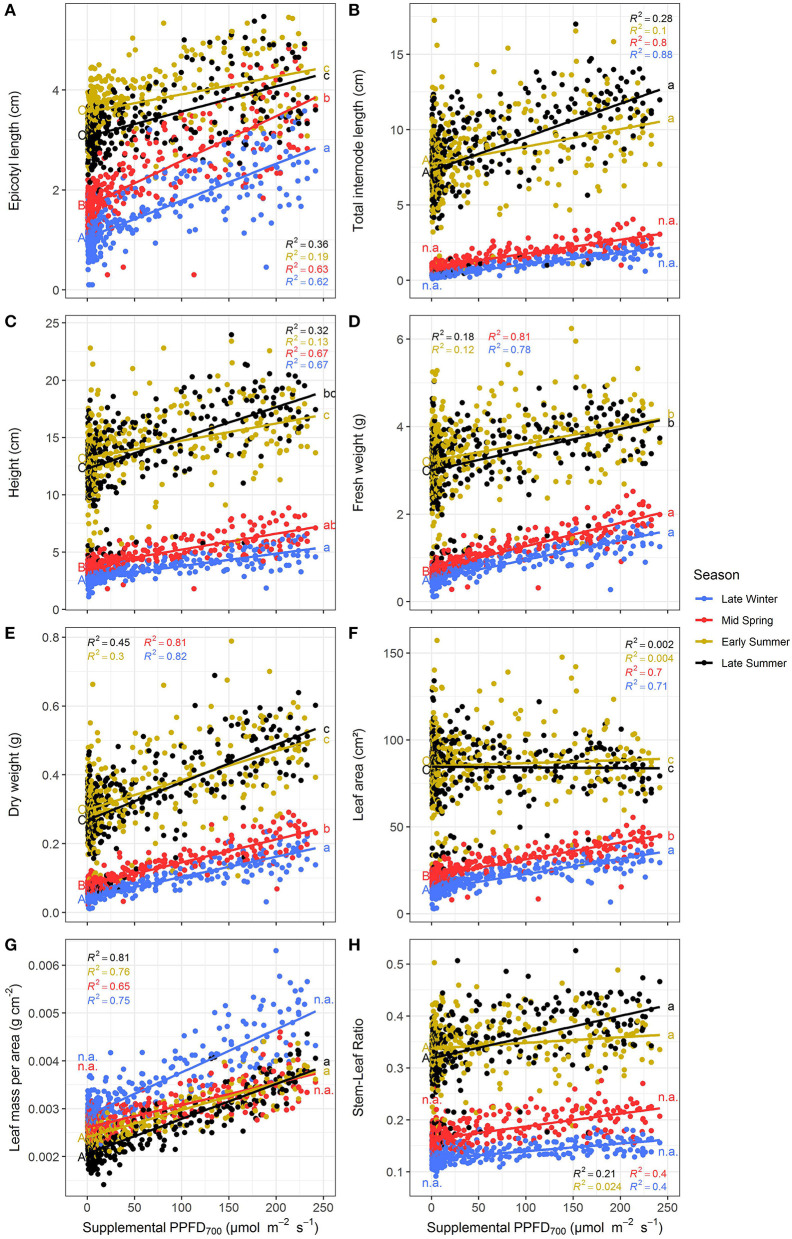
Effect of the season on epicotyl length **(A)**, total internode length **(B)**, height **(C)**, fresh weight **(D)**, dry weight **(E)**, leaf area **(F)**, leaf mass per area **(G)**, and stem-leaf ratio **(H)** of *Ocimum basilicum* L. cv. “Edwina” cultivated under greenhouse conditions. Different lower-case and upper-case letters indicate significant differences in slopes and intercepts between seasons (*p* < 0.05; n.a., non-estimable), respectively. Linear regression lines of each light color are indicated. Regressions are based on 120 plants per treatment, each with a specific light dose. Each data point shows an average of the three light colors, two harvest points and two experimental repetitions.

**Figure 6:**
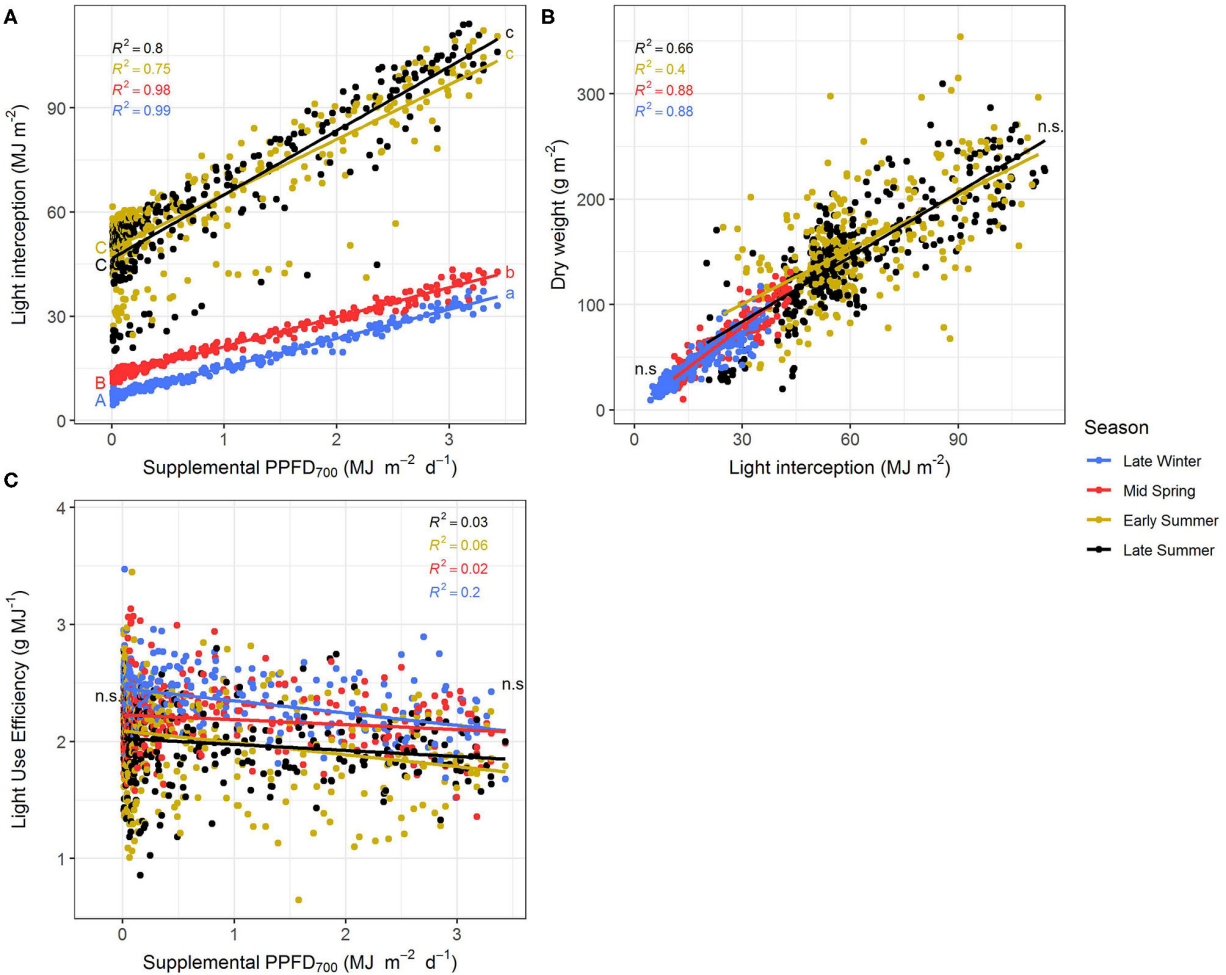
Effect of the season on light interception **(A)**, dry weight **(B)**, and light use efficiency **(C)** of *Ocimum basilicum* L. cv. “Edwina” cultivated under greenhouse conditions. Different lower-case and upper-case letters indicate significant differences in slopes and intercepts between seasons (*p* < 0.05; n.s., non-significant), respectively. Linear regression lines of each light color are indicated. Regressions are based on 120 plants per treatment, each with a specific light dose. Each data point shows an average of the three light colors, two harvest points, and two experimental repetitions.

The efficiencies (slopes) and magnitudes (intercepts) of all plant parameters showed significant (*p* < 0.05) differences between the four seasonal environments except for LUE and EUE (Supplementary Tables 1, 2). Efficiencies of the different light colors were not affected by the seasons. It could clearly be observed that the efficiencies of supplemental LED lighting were lower during early and late summer compared to winter and spring, whereas effect magnitudes were higher in summer than in winter (Figures 5, 6). Although the growing periods until intermediate (14–20 days) and final harvest (28–33 days) were similar in all seasons, environmental conditions inside the greenhouse largely differed between the experiments (Table 1). Temperatures during winter and spring experiments were roughly 5°C lower than in summer and natural day light integral (DLI) approximately doubled from 7.1 mol m^−2^ d^−1^ in late winter to 15.7 mol m^−2^ d^−1^ in early summer. Differences in natural DLI were associated with differences in SL/NL that decreased with an increase in natural light. Plants in summer were taller (Figures 5A–C) and had larger leaves (Figure 5F) compared to plants grown in winter and spring. Furthermore, plants invested roughly three to four times more dry matter into the stem than into the leaves during summer (Figure 5H). At the same time, lower LMA's were observed (Figure 5G). The increased amount of light during summer, coupled with the altered plant morphology was associated with increased light interception (Figure 6A) leading to an overall higher biomass production compared to winter and spring (Figures 5D,E). LUE was not affected by the seasons (Figures 6B,C) although it tended to be lower during both summer experiments (Figure 6C).

### Effect of PPFD_800_ on Supplemental LED Lighting Efficiencies

**Figure 7:**
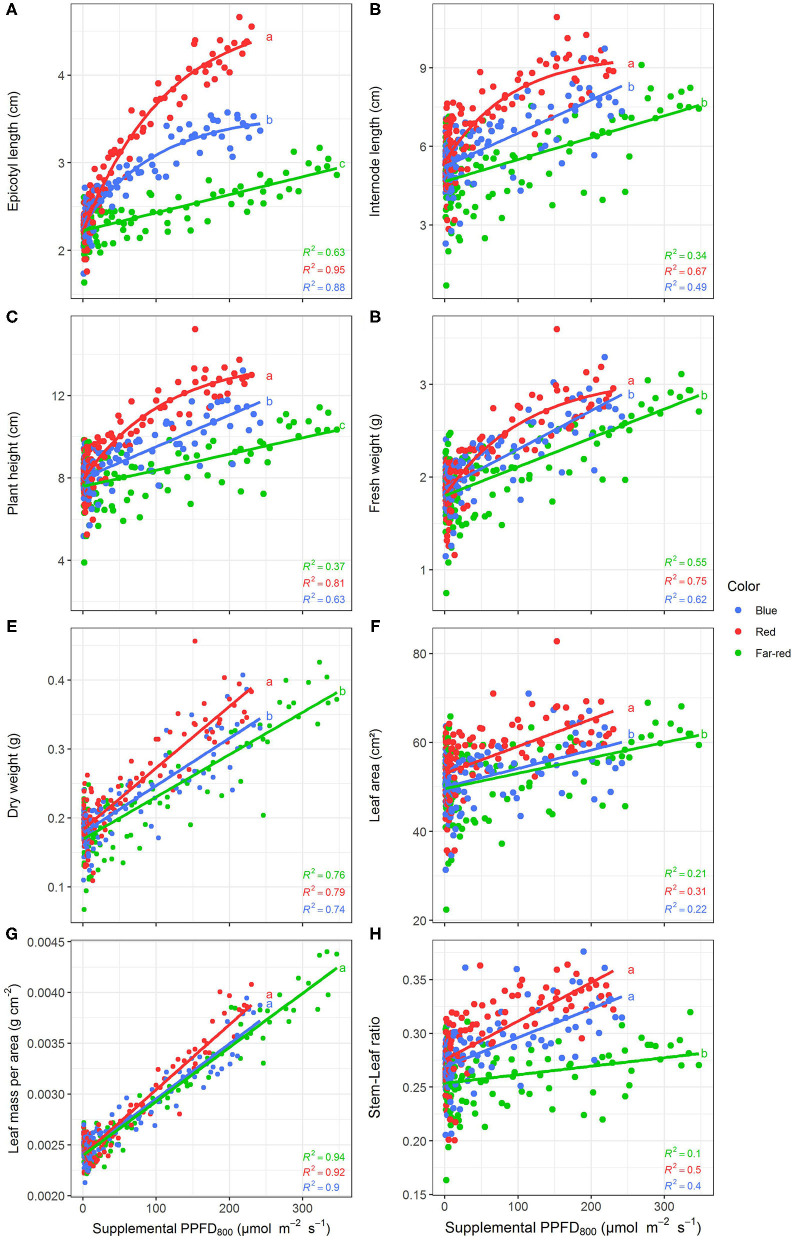
Effect of supplemental PPFD_800_ (400–800 nm) with different spectra on epicotyl length **(A)**, total internode length **(B)**, height **(C)**, fresh weight **(D)**, dry weight **(E)**, leaf area **(F)**, leaf mass per area **(G)**, and stem-leaf ratio **(H)** of *Ocimum basilicum* L. cv. “Edwina” cultivated under greenhouse conditions. Different lower-case letters indicate significant differences in slopes between colors (*p* < 0.05). Linear or monomolecular regression lines of each light color are indicated. Regressions are based on 120 plants per treatment, each with a specific light dose. Each data point shows an average of the four seasons, two harvest points and two experimental repetitions.

**Figure 8:**
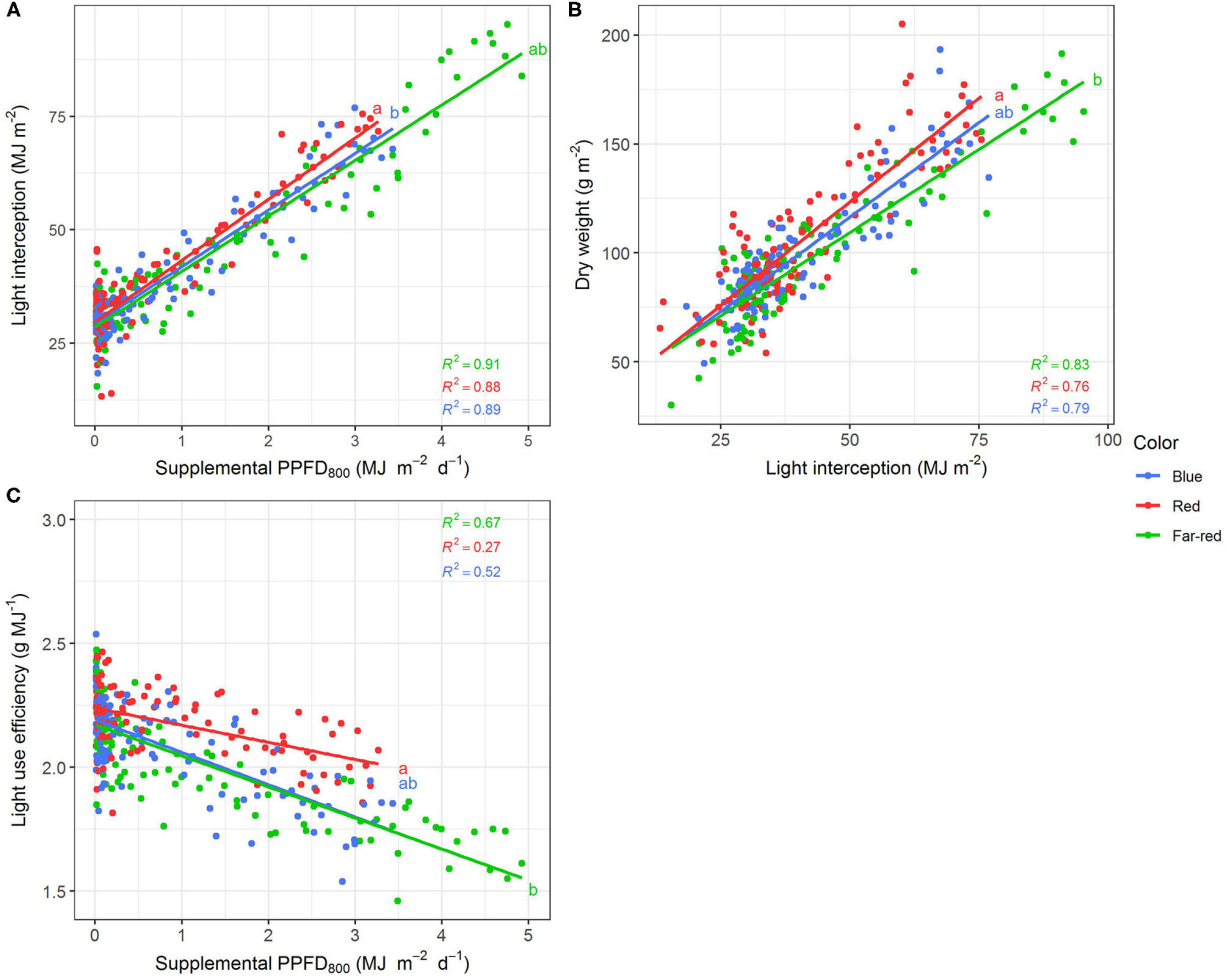
Effect of supplemental PPFD_800_ (400–800 nm) with different spectra on light interception **(A)**, dry weight **(B)** and light use efficiency **(C)** of *Ocimum basilicum* L. cv. “Edwina” cultivated under greenhouse conditions. Different lower-case letters indicate significant differences in slopes between colors (*p* < 0.05). Linear regression lines of each light color are indicated. Regressions are based on 120 plants per treatment, each with a specific light dose. Each data point shows an average of the four seasons, two harvest points and two experimental repetitions.

Efficiencies of supplemental LED lighting were significantly altered when dry matter was related to PPFD_800_ (Figures 7, 8) instead of PPFD_700_ (Figures 3, 4). Lighting efficiency on plant dry weight production was strongly reduced when far-red was considered photosynthetic active (Figure 7E). Changes in dry weight production were mainly linked to a severely reduced LUE (Figures 8B,C) rather than light interception (Figure 8A) under PPFD_800_ compared to PPFD_700_. The lower LUE was primarily associated with altered morphological traits related to photosynthesis such as leaf area (Figure 7F) and LMA (Figure 7G).

## Discussion

Efficiencies of LED lighting on plant growth and morphology were determined under the same amount of supplemented PPFD_700_ in all light treatments to allow a clear distinction between supplemental light quality and quantity effects. Light spectra and natural environments (seasons) had significant effects on the efficiencies. However, the different light spectra lead to unexpected plant responses. Furthermore, considering PPFD_800_ instead of PPFD_700_ resulted in reduced efficiencies. Possible causes and implications are discussed below.

### Significance of the Light Color on the Plants Adaption Strategies to Light

It was striking that, contrary to our expectations and literature on basil cultivars “Eleonora” and “Emily” (Dörr et al., 2020, Larsen et al., 2020) the plants in our study did not show typical shade avoidance reactions, but had increased stem elongation under red light. Morgan and Smith (1979) showed that stem extension rate and petiole length are negatively related to an increase in phytochrome photostationary state (number of active phytochromes to total phytochromes) in most species. However, the species *Oxalis acetosella* L. exhibited a clear opposite response in their study indicating a species-specific behavior. Moreover, it has been shown that cultivars of the same species may react differently toward the light quality under that they are grown (e.g., Ohashi-Kaneko et al., 2006). Consequently, the observed reaction to R/FR seems to be a cultivar-specific rather than a species-specific response.

It was reported that during early photomorphogensis of tomato seedlings monochromatic red light stimulated plant elongation whereas monochromatic blue light induced a more compact size (Izzo et al., 2020). Our results concur with this observation during early plant growth as plants in our trials were already more elongated and had higher leaf areas under red light than under far-red and blue light during the intermediate harvest (data not shown). Moreover, reducing the plant density under the same experimental setup resulted in less elongated plants endorsing the above suggested line of thought (Supplementary Figure 1). Hence, we presume that the stimulated early seedling growth under red light had resulted in an earlier canopy closure compared to the other light treatments which, as a consequence, lead to early competition for light and thus further facilitated plant elongation growth.

Another interesting aspect is that stem elongation was also more promoted by blue light than by far-red light. There are reports ranging from promotion (Heo et al., 2002; Johnson et al., 2020; Larsen et al., 2020) to reduction (Hoenecke et al., 1992; Islam et al., 2012, Izzo et al., 2020) of elongation growth by blue light. It was suggested that promoted plant elongation by blue light is a shade avoidance response which is attributed to lower phytochrome activity (Kong et al., 2018). Moreover, the elongation response to blue light varies among plant species (Kong et al., 2018, Johnson et al., 2020). Larsen et al. (2020) found promotion of stem elongation in basil under 90 and 100% blue light during the production in a vertical farm which was most likely related to reduced phytochrome activity. Hence, the observed increased elongation under blue light was likely related to reduced phytochrome activity in our study as well.

### Light Dose Effects on Growth Efficiency of Supplemental Radiation

The effects of light quality on plant growth and morphology in this study are striking. The high efficiency of red light in promoting stem elongation and leaf area was associated with significantly increased light interception compared to the other light quality treatments, and thereby contributed to the high efficiency of plant biomass production. An increase in leaf area and stem length is more favorable for light capture due to a higher light penetration in the canopy and increased light absorption (Sarlikioti et al., 2011). Blue light was the least efficient in promoting biomass. An increase in blue light fraction is often associated with a decreased leaf area (Hernández and Kubota, 2014, 2016; Kaiser et al., 2019; Larsen et al., 2020) and radiation capture rather than changes in net-assimilation per unit leaf area (Snowden et al., 2016). In our study, however, the lower biomass of plants grown under blue LEDs appears to be mainly a result of the lower LUE instead of effects on light capture even though light interception was quite low as well. Although blue light is essential for proper functioning of photosynthesis (Hogewoning et al., 2010; Trouwborst et al., 2016), it is less efficiently used for photosynthesis in leaves (McCree, 1972; Hogewoning et al., 2012). The low LUE was accompanied by a low LMA under blue light. Increasing proportions of blue light are usually associated with the development of sun-type leaf characteristics with high LMA and high photosynthetic capacity (e.g., Buschmann et al., 1978; Lichtenthaler et al., 1980; Matsuda et al., 2008; Kaiser et al., 2019). However, increasing proportions of blue light did not affect LMA in rice (Ohashi-Kaneko et al., 2006) and basil (Larsen et al., 2020). Hence, the low LMA likely explains the lower light utilization by the leaves. The aforementioned train of thought that the observed changes in LUE are related to altered leaf morphology that affect leaf photosynthetic capacity (Oguchi et al., 2003), and in our case that the low LMA may explain the lower LUE under blue light, is consistent with the observed increase in LMA and LUE in the far-red light treatment. Increasing LMA was previously associated with a higher far-red fraction in the light source (Dörr et al., 2020) and increasing far-red light dose (Larsen et al., 2020) in basil although an opposite response toward an increase in far-red photons or lowered R/FR was reported in other species (Kalaitzoglou et al., 2019; Zou et al., 2019).

### Importance of Seasonality on Supplemental LED Lighting Effects

The variation in solar DLI (Table 1) mainly explains the observed seasonal differences in growth and morphology. Plants grown in winter were not only significantly shorter, but also had lower leaf area and weight compared to plants cultivated in summer which can be explained by reduced rates of photosynthesis (Chang et al., 2008; Dou et al., 2018). Dou et al. (2018) suggested a DLI of 12.9 mol m^−2^ d^−1^ for optimal commercial production of basil in indoor farming. This suggests that the summer sets were operating largely under light saturation, explaining the lack of differences between the two sets. Temperature was likely the most important factor for variation in LMA. It has been demonstrated that a decrease in LMA is a common response to elevated temperature (Poorter et al., 2009a,b; Poorter et al., 2010) which was observed in basil as well (Chang et al., 2005; Walters and Currey, 2019). The seasonal variation in dry mass production in our trials was rather a consequence of changes in DLI than of light utilization efficiency, as it could be expected from the decreased LMA. The altered plant architecture was more favorable for light interception and whole-canopy photosynthesis (Buck-Sorlin et al., 2011; Sarlikioti et al., 2011; Song et al., 2013; Chen et al., 2014).

### Impact of Supplemental PPFD_800_ Under Solar Radiation

Latest studies revised the Emerson enhancement effect (Emerson et al., 1957) showing that far-red photons have positive, synergetic effects on photosynthesis in combination with shorter wavelength (Hogewoning et al., 2012; Zhen and van Iersel, 2017; Murakami et al., 2018; Zhen et al., 2018; Kono et al., 2020). Besides the positive effect on photosynthesis it was even demonstrated that far-red photons have equal photon efficiency in combination with shorter wavelengths (Zhen and Bugbee, 2020a) and it was thus suggested that far-red photons (701–750 nm) should be included in the definition of PAR (Zhen and Bugbee, 2020a,b). As it is to be expected, efficiencies in the far-red treatment decreased considerably when growth and morphological traits were related to PPFD_800_ instead of PPFD_700_ as the amount of total photons was far greater in the far-red light regime compared to the others. Parameters associated with photosynthesis such as dry weight, LMA, biomass partitioning and LUE were particularly affected. Hence, for future investigations it would be interesting to compare efficiencies of different light regimes with the same PPFD_800_.

### Perspectives and Implications of the Efficiency of Supplemental LED Lighting for Future Applications

Greenhouse production in northern latitudes is mainly limited by low incident light and short photoperiods during winter. Thus, supplemental lighting has been used to enable a year-round production of crops (Davis and Burns, 2016). Our results add to this demonstrating that the efficiency of supplemental LED lighting is significantly affected by seasonal variations. Efficiencies were significantly higher during low light conditions (Late Winter and Mid Spring) compared to high light conditions (Early and Late Summer) and associated with changes in the ratio of supplemental to natural light. A more precise application of supplemental lighting taking SL/NL into account may contribute to energy saving and cost-reduction during greenhouse cultivation under solar radiation. Hence, we suggest that it may be used as an indicator to assess and evaluate supplemental lighting effects under solar background radiation, and would allow a better comparison among research studies.

## Conclusions

The present study demonstrated that there are striking differences in the efficiencies among light qualities highlighting the importance of the choice of the light color during greenhouse production and the plants strategy to cope with the growing environment. Plant responses to the different light qualities were mainly associated with two adaptation strategies: increased light interception due to a plant architecture more favorable for light capture under red light and changed LUE due to altered leaf morphology under far-red light. Blue light was the least efficient color in affecting plant growth and morphology. However, efficiencies were significantly reduced when PPFD_800_ was considered instead of PPFD_700_. Furthermore, our results underline the significance of the natural growth environment (seasons) on the efficiency of supplemental LED lighting as indicated by the altered efficiencies with changing natural light conditions. Seasonal changes in biomass production were attributed to increased light interception due to altered crop architecture more favorable for light absorption rather than changes in light use efficiency. Finally, it is suggested that the ratio of supplemental to natural light is a good indicator to quantitatively evaluate plant responses to supplemental LED lighting during greenhouse production throughout the year.

## Data Availability Statement

The raw data supporting the conclusions of this article will be made available by the authors, without undue reservation.

## Author Contributions

This study was designed by JS, AF, and HS. JS acquired the data, performed the analyses, drafted the manuscript, and discussed with AF and HS. All authors revised the article and approved the final version to be submitted.

## Conflict of Interest

The authors declare that the research was conducted in the absence of any commercial or financial relationships that could be construed as a potential conflict of interest.
