# Supplementary material for: Seasonal Efficiency of Supplemental LED Lighting on Growth and Photomorphogenesis of Sweet Basil
Source: Front Plant Sci. 2021 Apr 6;12:609975. doi: 10.3389/fpls.2021.609975 (PMC8056084; doi:10.3389/fpls.2021.609975)
Supplement: Supplementary file 1 [file Table_1.docx]

Supplementary Material

**Table S1** Effect of light color, season and harvest on the slopes of the regressions (i.e. efficiencies) of supplemental LED lighting of growth and morphological traits of *Ocimum basilicum* L. cv. 'Edwina' cultivated under greenhouse conditions. P-values were derived from ANOVA for all three factors and their two-way interactions.

|  | **p-values** | | | | | |
| --- | --- | --- | --- | --- | --- | --- |
| **Parameter** | **Color** | **Season** | **Harvest** | **Color:Season** | **Color:Harvest** | **Season:Harvest** |
| **Epicotyl** | < 0.001 | < 0.001 | < 0.001 | n.s. | n.s. | < 0.01 |
| **Internode** | < 0.001 | < 0.001 | < 0.05 | n.s. | n.s. | n.s. |
| **Height** | < 0.001 | < 0.01 | < 0.01 | n.s. | n.s. | < 0.01 |
| **Fresh weight** | < 0.05 | < 0.001 | n.s. | n.s. | n.s. | n.s. |
| **Dry weight** | < 0.01 | < 0.001 | < 0.05 | n.s. | n.s. | < 0.05 |
| **Leaf area** | < 0.05 | < 0.001 | < 0.01 | n.s. | n.s. | n.s. |
| **LMA** | < 0.01 | < 0.05 | n.s. | n.s. | n.s. | n.s. |
| **Stem-Leaf ratio** | < 0.001 | < 0.05 | n.s. | n.s. | n.s. | n.s. |
| **Light interception** | < 0.01 | < 0.001 | n.s. | n.s. | n.s. | n.s. |
| **Dry weight m^-2^** | < 0.05 | n.s. | < 0.01 | n.s. | n.s. | n.s. |
| **LUE** | < 0.05 | n.s. | < 0.05 | n.s. | n.s. | n.s. |
| **EUE** | n.s. | n.s. | < 0.05 | n.s. | n.s. | n.s. |

**Table S2** Effect of light color, season and harvest on the intercepts of the regressions (i.e. magnitude) of supplemental LED lighting of growth and morphological traits of *Ocimum basilicum* L. cv. 'Edwina' cultivated under greenhouse conditions. P-values were derived from ANOVA for all three factors and their two-way interactions.

|  | **p-values** | | | | | |
| --- | --- | --- | --- | --- | --- | --- |
| **Parameter** | **Color** | **Season** | **Harvest** | **Color:Season** | **Color:Harvest** | **Season:Harvest** |
| **Epicotyl** | n.s. | < 0.001 | < 0.001 | n.s. | n.s. | < 0.01 |
| **Internode** | n.s. | < 0.001 | < 0.001 | n.s. | n.s. | n.s. |
| **Height** | < 0.05 | < 0.001 | < 0.001 | n.s. | < 0.05 | < 0.01 |
| **Fresh weight** | n.s. | < 0.001 | < 0.001 | n.s. | n.s. | < 0.05 |
| **Dry weight** | n.s. | < 0.001 | < 0.001 | n.s. | n.s. | < 0.05 |
| **Leaf area** | n.s. | < 0.001 | < 0.001 | n.s. | n.s. | < 0.01 |
| **LMA** | n.s. | n.s. | < 0.05 | n.s. | n.s. | n.s. |
| **Stem-Leaf ratio** | n.s. | < 0.001 | < 0.001 | n.s. | n.s. | < 0.05 |
| **Light interception** | < 0.05 | < 0.001 | < 0.001 | n.s. | n.s. | < 0.001 |
| **Dry weight m^-2^** | n.s. | n.s. | < 0.01 | n.s. | n.s. | < 0.05 |
| **LUE** | n.s. | n.s. | < 0.001 | n.s. | n.s. | < 0.05 |
| **EUE** | < 0.001 | n.s. | < 0.001 | n.s. | n.s. | < 0.05 |


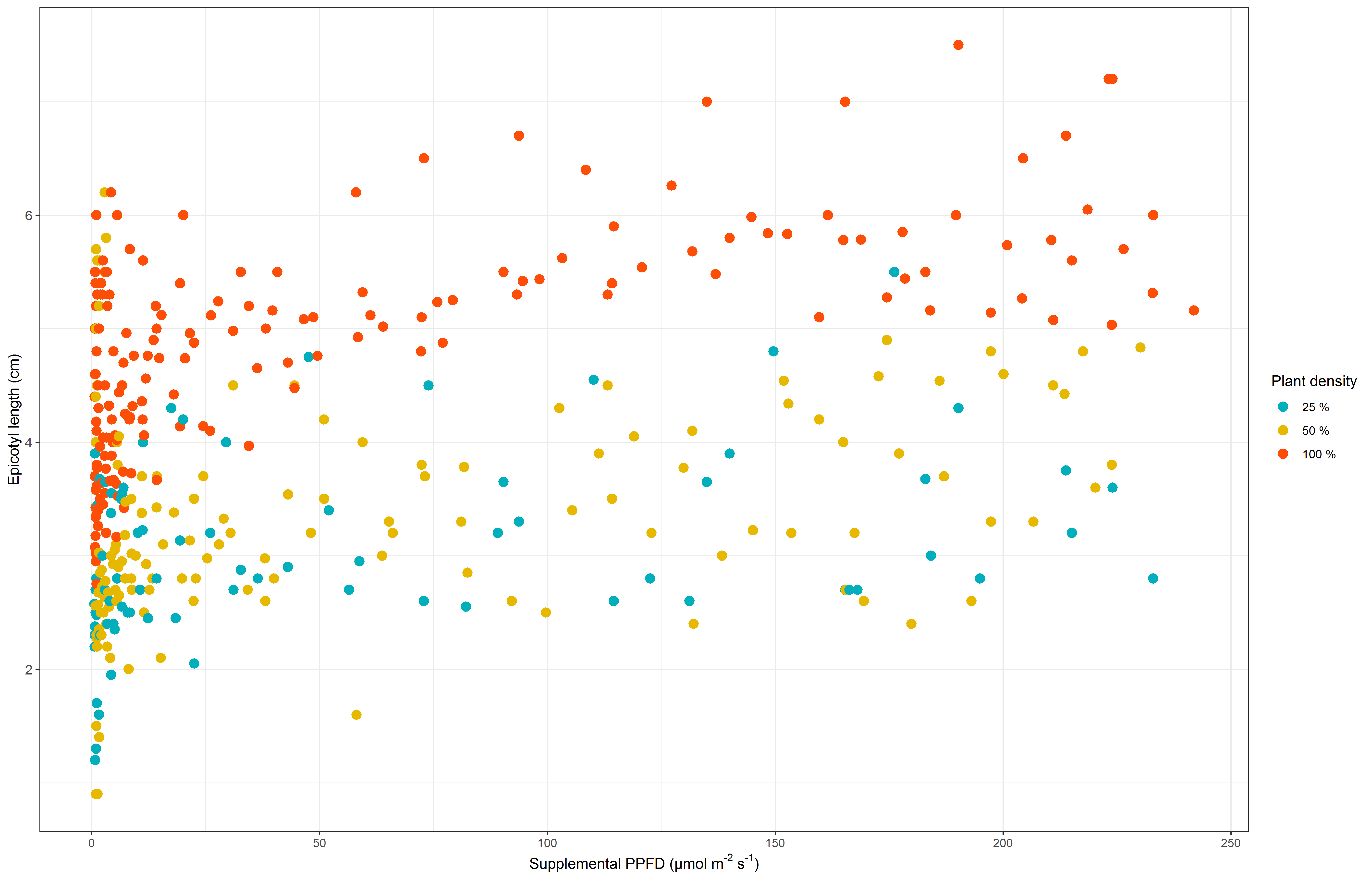


Figure S1 Effect of plant density on epicotyl length of *Ocimum basilicum* L. cv. 'Edwina' cultivated under red LEDs. A plant density of 100 % equaled the density used in the present study.
